# Hydrogen-Rich Water Ameliorates Murine Chronic Graft-versus-Host Disease through Antioxidation

**DOI:** 10.1155/2021/1165928

**Published:** 2021-10-14

**Authors:** Liren Qian, Jiaxin Liu, Weina Ma, Yu Liu, Xiaona Wang, Daihong Liu

**Affiliations:** Senior Department of Hematology, The Fifth Medical Center, Chinese PLA General Hospital, Chinese PLA Medical School, Beijing, China

## Abstract

**Background:**

Allogeneic hematopoietic stem cell transplantation (allo-HSCT) is an important treatment option for various hematopoietic diseases and certain hereditary diseases. Chronic graft-versus-host disease (cGVHD) has become the main life-threatening complication and cause of death in later stage postallo-HSCT. Current treatment options for cGVHD are limited. Hydrogen gas (H_2_) has been demonstrated that has antioxidative, anti-inflammatory, and antifibrosis effects. The aim of this study was to confirm whether oral administration hydrogen-rich water exerted therapeutic effects on a scleroderma cGVHD mouse model and tried to explain the mechanism underly it.

**Methods:**

A mouse cGVHD model was established by haploidentical bone marrow transplantation. To evaluate therapeutic effects of H_2_ on cGVHD, survival rate, changes in clinical scores, and skin pathologic characteristics of cGVHD mice were observed. To evaluate its therapeutic mechanism, we detected the expression levels of antioxidative enzymes heme oxygenase-1(HO-1) and NAD (P)H: quinone acceptor oxidoreductase 1(NQO1) in skin homogenates. We also detected the expression level of the apoptotic protein caspase-3 in skin homogenates.

**Results:**

1-month survival rate of cGVHD mice in the hydrogen group reached 93.3%, significantly higher than 66.7% in the nonhydrogen group (*p* < 0.05). Clinical score of cGVHD mice was improved by hydrogen-rich water at 96 days posttransplantation (2.2 versus 4.5, *p* < 0.05). The skin pathological condition of cGVHD mice was significantly improved by hydrogen-rich water. At 96 days posttransplantation, average skin pathological hematoxylin and eosin (HE) staining score in the hydrogen group was 1.05, which was significantly lower than 3.2 in the nonhydrogen group (*p* < 0.01). Average Masson staining score was 0.6 point in the hydrogen group, lower than 0.9 point in the nonhydrogen group (*p* < 0.05). Both the relative expression levels of HO-1 and NQO1 proteins in skin specimens of cGVHD mice in the hydrogen group were lower than that in the nonhydrogen group (2.47 versus 6.21 and 1.83 versus 3.59, *p* < 0.05). The relative expression level of caspase-3 protein in skin specimens of cGVHD mice increased to 7.17 on the 96th day after transplantation, significantly higher than 4.36 in the hydrogen group.

**Conclusion:**

In this study, we found that oral hydrogen-rich water improved the survival rate and clinical symptoms of cGVHD mice by antioxidant and antiapoptosis. This study would pave the way for further clinical study, which may provide a new treatment option for cGVHD.

## 1. Introduction

Allogeneic hematopoietic stem cell transplantation (allo-HSCT) is an important treatment option for various hematopoietic diseases and certain hereditary diseases. Chronic graft-versus-host disease (cGVHD) has become the main life-threatening complication and cause of death in later stage postallo-HSCT [1, 2]. With the decrease in early mortality posttransplantation, the increasing upper age limit of recipients, and the widespread application of unrelated donors and peripheral blood hematopoietic stem cells, the incidence of cGVHD has gradually increased [3, 4]. 2-year cumulative incidence of cGVHD posttransplantation that needs treatment was 30%-40% [2]. Glucocorticoids with or without calcineurin inhibitors (CNI) were always used as the initial treatment of cGVHD in the past few decades [2]. However, glucocorticoids may cause serious adverse effects after long-term application, including severe infections, peptic ulcers, femoral head necrosis, osteoporosis, diabetes, and hypertension [5]. Besides, during the tapering of glucocorticoids, cGVHD symptoms often relapse or even aggravate [5]. If the disease relapses or progresses, second-line treatment is often required. 50%-60% cGVHD patients need second-line therapy [6, 7]. However, there is currently no standard preferred second-line treatment [8]. Although some new drugs such as ruxolitinib have brought unprecedented curative effects in this field in recent years [9–11], clinical trials are still encouraged, and a better therapy method is in urgent need for cGVHD [12].

In 2007, Ohsawa et al. systematically confirmed the free radicals scavenging ability of hydrogen [13]. They found that H_2_ had similar therapeutic effects in a cerebral ischemia-reperfusion injury rat model by scavenging hydroxyl radicals (·OH) comparing with tacrolimus. Besides to its free radical scavenging ability, researchers also found that hydrogen has anti-inflammatory effects in autoimmune hepatitis [14], systemic inflammatory response syndrome [15], inflammatory bowel disease [16], allergic dermatitis [17], lipopolysaccharide- (LPS-) induced paw edema [18], and other animal inflammatory disease models, which is also similar to tacrolimus. Moreover, H_2_ has antifibrosis effects. It was found that breathing 4% H_2_ significantly delayed the progression of pulmonary fibrosis in a radiation induced pulmonary fibrosis model [19]. They confirmed that H_2_ significantly reduced the fibrotic lesions in the lungs of mice. The main pathophysiological process of cGVHD is immunoinflammatory responses, and the characteristic pathological change is fibrosis [12, 20, 21]. Oxidative stress, inflammation imbalance, and fibrosis play important roles in the progression of cGVHD [12, 20, 21]. Therefore, we speculated that H_2_ may exert potential therapeutic effects on cGVHD after allo-HSCT. In this study, an attempt was made to confirm whether oral administration hydrogen-rich water exerted therapeutic effects on a scleroderma cGVHD mouse model and tried to explain the mechanism underly it.

## 2. Materials and Methods

### 2.1. Hydrogen-Rich Water Production

Hydrogen-rich water was produced by dissolving hydrogen in sterile drinking water for 6 hours under high pressure (0.4 MPa) to a supersaturated level as we previously reported [22–24]. Hydrogen-rich water was freshly prepared every 12 hours, which ensured that a concentration of more than 0.6 mmol/L was maintained. Gas chromatography (Biogas Analyzer Systems-1000, Mitleben, Japan) was used to confirm the content of hydrogen in saline by the method described by Ohsawa et al. [13].

### 2.2. Mice

All the protocols were approved by the Chinese PLA General Hospital in accordance with the Guide for Care and Use of Laboratory Animals published by the US NIH (publication No. 96-01). Female C57BL/6 N mice and male B6D2F1 mice were obtained from Beijing Vital River Laboratory Animal, Inc. (Beijing, China, http://www.vitalriver.com.cn). All mice were studied at between 10 and 12 weeks of age. Mice were housed in autoclaved cages with sterile food and water.

### 2.3. Chronic Graft-versus-Host Disease (cGVHD) Model

A cGVHD model was established as previously described [25]. B6D2F1 mice received total body irradiation (TBI) on day 1 (5.5 Gy, two doses on the same day, with an interval of 3-4 hours). On day 0, the control group was injected with 5 × 10^6^ T cell–depleted bone marrow cells (TCD-BM) of C57BL/6 mice through the tail vein. In the cGVHD model group, 5 × 10^6^ TCD-BM plus purified 1 × 10^6^ splenic T cells of C57BL/6 mice were injected to irradiated B6D2F1 mice. In the hydrogen group, mice were given hydrogen-rich water from the 8^th^ day posttransplantation when the mice have cGVHD symptoms until they were sacrificed. In the control group and nonhydrogen group, mice were routinely fed with sterile water.

### 2.4. Survival Assays

After transplantation, the mice were returned to individually ventilated cages and routinely cared. Their survival status was observed daily, and the survival was checked and recorded for 30 days.

### 2.5. Evaluation of cGVHD

Chronic GVHD symptoms of mice are clinically scored every five days after transplantation, mainly from the following 5 aspects [26]: weight loss (scored 0: <10%; scored 1: 10%-25%; scored 2: >25%), activity (scored 0: normal; scored 1: mild to moderately decreased; scored 2: stationary unless stimulated), posture (scored 0: normal; scored 1: hunching only at rest; scored 2: severe hunching impairs movement), fur texture (scored 0: normal; scored 1: mild to moderate ruffling; scored 2: severe ruffling/poor grooming), and skin integrity (scored 0: normal; scored 1: incomplete paw/tail scales; scored 2: obvious areas of denuded skin). The scores of these five aspects were added together to evaluate the severity of cGVHD.

### 2.6. Tissue Histopathology

About 2 cm^2^ shaved skin from interscapular region was selected. The hematoxylin and eosin (HE) staining was performed as we previously described [27]. A dermatologist, blinded to the groups of animals, scored from five aspects: epidermal structural changes, inflammatory cell infiltration, reduction or loss of hair follicles, dermal fibrosis, and reduction or loss of fat. Each index is rated as 0-2 points according to the severity of the lesion. The total score is between 0 and 10 points [28]. Masson staining was also performed according to the previous literature [23], and scores were given according to the thickness and looseness of collagen fibers: 0 (normal), 0.5 (minor), 1 (mild), 2 (moderate), and 3 (severity). The skin of each group was scored for pathology 96 days after transplantation.

### 2.7. Western Blot

The specimens of skin tissue were collected and lysed as previously described [29]. The skin samples were collected and frozen in dry ice and stored at -70°C until assayed by WB analysis. We homogenized the skin specimens on ice by sonication and dissolved in lysis buffer, which contains phosphate-buffered saline (PBS, pH 7.4), 1% Tergitol NP-40 (Sigma-Aldrich, St. Louis, MO), 0.5% sodium deoxycholate (Sigma), 1% sodium dodecyl sulfate (SDS) (Sigma), 1 mM EDTA (Sigma), 1 mM EGTA (Sigma), 1% protease inhibitor cocktail (Sigma), and 0.6 mM phenylmethanesulfonyl fluoride (PMSF). Then, the homogenate was centrifuged at 14,000 rpm for 30 minutes at 4°C [30]. Protein concentrations were detected by NanoDrop 1000 spectrophotometer (Thermo Fisher Scientific) [31]. The expression levels of HO-1, NQO1, and caspase-3 proteins in the skin tissues of different groups were detected by western blot analysis as previously described [32]. In the western blot analysis, we obtained the following antibodies from Cell Signaling Technology: anti-HO-1, anti-NQO1, anti-caspase-3, and anti-*β*-actin.

## 3. Results

### 3.1. Therapeutic Effects of Hydrogen on cGVHD Mice

#### 3.1.1. Hydrogen Increased the Survival Rate of cGVHD Mice

**Figure 1:**
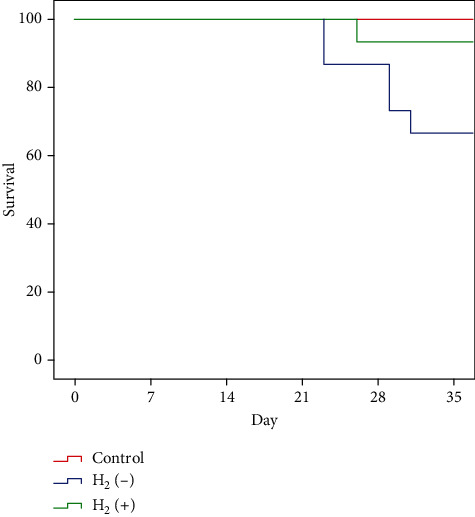
The survival rate of chronic GVHD mice in the hydrogen group was significantly higher than that in the nonhydrogen group (*p* < 0.05).

Oral giving more than 2 weeks of hydrogen-rich water improved the survival rate of cGVHD mice (Figure 1). The 30-day survival rate of cGVHD mice in the hydrogen water group was 93.3%, significantly higher than that in the nonhydrogen cGVHD group (66.7%, *p* < 0.05).

#### 3.1.2. Hydrogesgen Improved cGVHD Mice Clinical Symptoms

**Figure 2:**
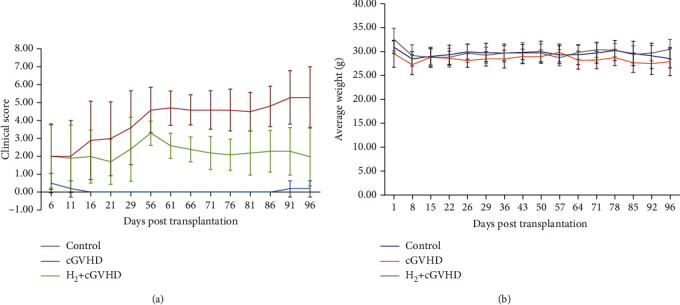
With the time of drinking H_2_-rich water increased, the clinical improvement of chronic GVHD mice becomes more obvious. At 96 days after transplantation, the clinical score of chronic GVHD mice in the hydrogen group was significantly lower than that in the nonhydrogen group (Figure 2(a), *p* < 0.05). The weight of chronic GVHD mice in the hydrogen group was not statistically different from that in the nonhydrogen group (Figure 2(b), *p* > 0.05).

Compared with the nonhydrogen group, the clinical symptoms of the mice in the hydrogen group began to improve after drinking hydrogen-rich water for one week (Figure 2(a)). With the time of drinking hydrogen-rich water increased, the improvement of clinical symptoms becomes more obvious. At 96 days posttransplantation, average clinical score of the cGVHD mice in the hydrogen group was 2.0 points, which was less than that in the nonhydrogen water group (5.3 points, *p* < 0.05). The average body weight of cGVHD mice in the hydrogen group was higher compared with that of the nonhydrogen water group without statistical difference (30.50 g vs. 27.92 g, *p* > 0.05, Figure 2(b)).

#### 3.1.3. Hydrogen Improved cGVHD Mice Skin Pathology

**Figure 3:**
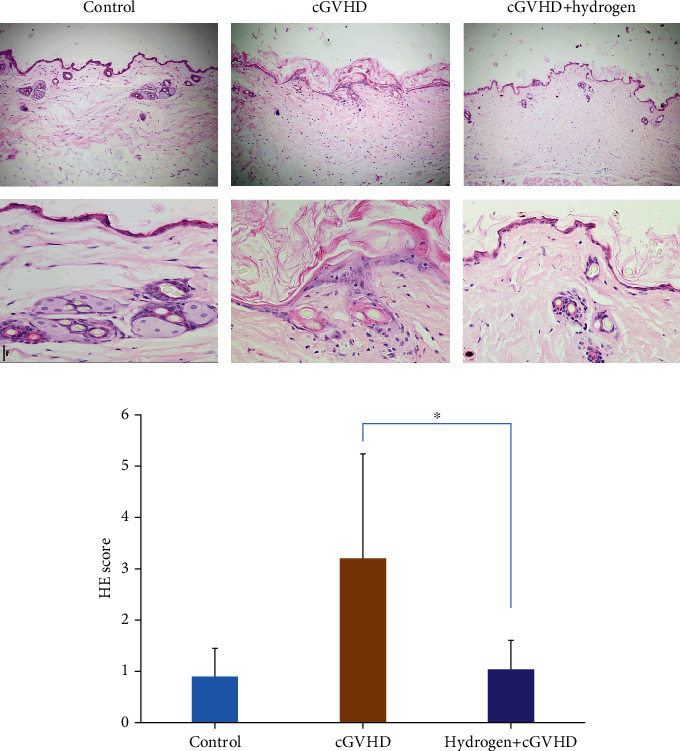
The skin pathological HE staining score of chronic GVHD mice in the hydrogen group was significantly lower than that in the nonhydrogen group (*p* < 0.01).

**Figure 4:**
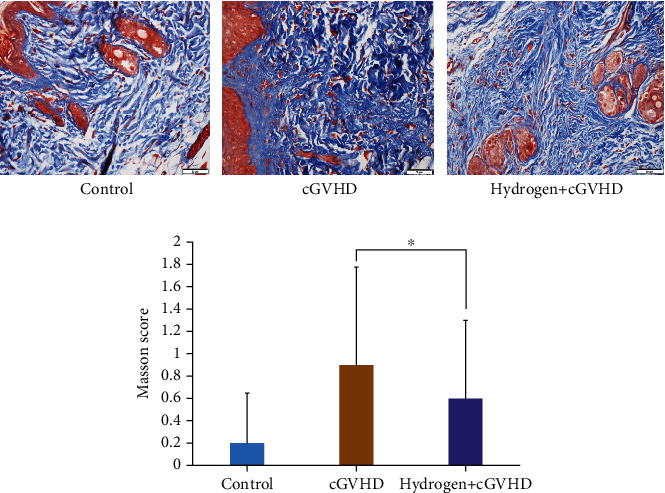
The skin pathological Masson staining fibrosis score of chronic GVHD mice in the hydrogen group was significantly lower than that in the nonhydrogen group (*p* < 0.05).

The skin pathological condition of cGVHD mice has been significantly improved after given hydrogen-rich water. At 96 days posttransplantation, the average skin pathological HE staining score in the hydrogen group was 1.05, significantly lower than 3.2 in the nonhydrogen group (*p* < 0.01, Figure 3). The average Masson staining score was 0.6 point in the hydrogen group, also lower than 0.9 point in the nonhydrogen group (*p* < 0.05, Figure 4).

### 3.2. The Mechanism of Hydrogen on cGVHD Mice

#### 3.2.1. Hydrogen Reduced the Expression Level of HO-1 and NQO1

**Figure 5:**
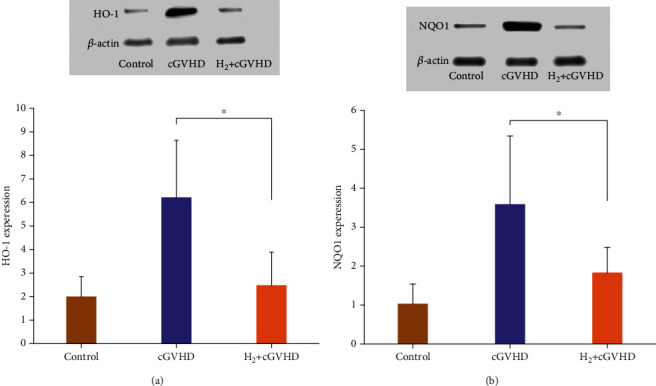
The relative expression level of HO-1 protein in the skin tissue of chronic GVHD mice in the hydrogen group was significantly lower than that in the nonhydrogen group (Figure 5(a), *p* < 0.05). The relative expression level of NQO1 protein in the skin tissue of chronic GVHD mice in the hydrogen group was significantly lower than that in the nonhydrogen group (Figure 5(b), *p* < 0.05).

The relative expression level of HO-1 protein (/*β*-actin) in the skin tissue of cGVHD mice in the hydrogen group was 2.47, which was significantly lower than 6.21 in the skin tissue of the nonhydrogen group cGVHD mice (Figure 5(a)). We found that the relative expression of NQO1 protein(/*β*-actin) in the skin tissue of cGVHD mice in the hydrogen group was 1.83, which was significantly lower than 3.59 in the nonhydrogen group (Figure 5(b)).

#### 3.2.2. Hydrogen Reduced the Expression Level of Caspase-3

**Figure 6:**
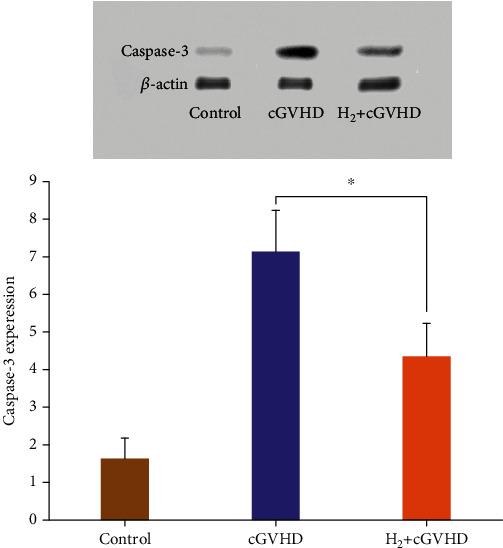
The relative expression level of caspase-3 protein in the skin tissue of chronic GVHD mice in the hydrogen group was significantly lower than that in the nonhydrogen group (*p* < 0.05).

The relative expression of caspase-3 protein (/*β*-actin) in the skin tissue of cGVHD mice significantly increased to 7.17 on the 96th day after transplantation, which was much higher than 4.36 in the hydrogen group, suggesting that molecular hydrogen significantly reduced the relative expression of apoptotic protein, which indicated that hydrogen has antiapoptotic ability by reducing the expression of caspase-3 protein (Figure 6).

## 4. Discussion

To our knowledge, this is the first study that proved oral saturated hydrogen-rich water has therapeutic effects on cGVHD in mice with scleroderma. It was confirmed that it exerted therapeutic effects by antioxidation and antiapoptosis. We demonstrated that hydrogen reduced the expression levels of HO-1 and NQO1 proteins in the cGVHD mice. We consider that hydrogen may neutralize oxygen free radicals and reduce the increased levels of HO-1 and NQO1 proteins caused by reactive oxygen species.

In our study, oral saturated hydrogen-rich water increased the 30-day survival rate of cGVHD mice by nearly 30%. The increase in the survival rate illustrated that hydrogen-rich water has therapeutic effects on mouse cGVHD as a whole. In addition, in this cGVHD animal model, we observed that oral hydrogen-rich water significantly improved the clinical symptoms of cGVHD in mice and improved the skin pathology of mice. Fibrosis has been proven playing an important role in the development of cGVHD disease [19]. Fibrosis leads to organ failure in patients with cGVHD, including scleroderma, bronchitis obliterans, and liver cirrhosis. Our research found that hydrogen-rich water mitigated the degree of skin fibrosis in cGVHD mice and improved the clincial symptoms of scleroderma in cGVHD mice. Formation of fibrosis often requires a relatively long-term process. Long-term oral hydrogen-rich water has no obvious toxic and side effects, making it can be used in a long-term. This feature is adapted to the long-term, repeated, and prolonged disease characteristics of cGVHD, which makes hydrogen-rich water very suitable for cGVHD. Previously, we have demonstrated that the survival rate of another cGVHD mice model was increased by intraperitoneally injecting hydrogen-rich saline, and the pathological changes in skin was also improved [27]. In this study, we used oral hydrogen-rich water, which is more convenient than hydrogen-rich saline for injection and inhalation of hydrogen gas.

HO-1 and NQO1 are two important antioxidant enzymes. HO-1 mainly catalyzes and decomposes heme into ferrous iron, carbon monoxide, and biliverdin and prevents the prooxidation effect of heme. Its byproduct bilirubin and reduced bilirubin exert effective antioxidative ability by scavenging free radicals [33, 34]. The expression level of HO-1 is significantly positively correlated with the levels of ROS. When the ROS levels in the body increased by various pathological conditions like hypoxia and acidosis, HO-1 would rise rapidly, playing a cytoprotective role in the body [34], which promoted heme catabolism and prevents induction of programmed cell death [35]. NQO1 is a cell-protecting antioxidant enzyme that exerts antioxidant effects from many aspects. NQO1 catalyzed quinone to hydroquinone, promoting the excretion of quinone. It also reduced quinones, quinoneimines, nitroaromatics, and azo dyes, thereby reducing the redox cycle to produce ROS, preventing oxidative damage [36]. Wefers et al. first confirmed that NQO1 has a direct antioxidant effect [37]. It was confirmed that NQO1 relies on the two-electron reduction mechanism, preventing quinone from participating in the oxidation cycle and generating active oxygen. As oxidative stress events occur in the body, NQO1 will also increase and play a protective role. Previously, we have confirmed that hydrogen regulated the levels of antioxidant enzymes superoxide dismutase (SOD), glutathione (GSH), and lipid oxidation product malondialdehyde (MDA) in the peripheral blood of mice injured by irradiation [22], exerting its radioprotective effects. In the current study, we found that in the skin tissues of mice, the expression levels of HO-1 and NQO1 proteins in the hydrogen group were significantly lower than those in the nonhydrogen cGVHD group. Many studies have confirmed that molecular hydrogen can directly react with oxygen free radicals such as hydroxyl radicals, thereby reducing oxidative damage [13, 19]. We believed that molecular hydrogen may reduce the oxidative stress level in cGVHD mice, thereby reducing the expression levels of HO-1 and NQO1 proteins in cGVHD mice.

Caspase-3 is one of the executioner caspases in apoptosis. It plays a vital role in cell apoptosis. It is responsible for cleaving most of the currently known apoptosis-related substrates. At the terminal of apoptosis, it is responsible for decomposing structural and regulatory proteins that shut down cell functions [38]. Chronic GVHD leads to apoptosis of tissue cells, resulting in a series of clinical manifestations. Our research found that the level of apoptosis protein caspase-3 in the molecular hydrogen group was significantly reduced. At present, we still consider molecular hydrogen exerting its antiapoptotic effect through antioxidation.

This research supplied novel ideas that were for treating cGVHD and has potential clinical application prospects, mainly due to the following points: first, H_2_ is nontoxic side effects and no residue in the body [39], which is adapted for the long-term, repeated, and prolonged disease characteristics of cGVHD. Second, hydrogen molecules have great penetrating ability because they are very small. They can quickly penetrate biological membranes and reach high concentration in cells to exert therapeutic effects [13]. Third, the price of H_2_ is low and easy to get. Although the current study confirmed that oral hydrogen-rich water has therapeutic effects on cGVHD and attempted to explain its mechanism, this research was limited confirming its therapeutic effect in a scleroderma cGVHD model. Chronic GVHD often involves multiple organs, including lungs, eyes, joints, gastrointestinal tract, and liver. As for whether hydrogen has therapeutic effects on cGVHD with other organs, it still needs to be further explored. Current studies on H_2_ have so far been mostly limited to animal or clinical observational researches. Current clear mechanism is its ability of scavenging free radicals. Its antiapoptotic and antifibrosis effects still mainly depended on its ability of scavenging free radicals. As for whether hydrogen gas is a signal molecule, the regulation of HO-1, NQO1, and caspase-3 protein expression levels through signal pathways still needs further research to confirm.

With the increasing incidence of cGVHD, it has become one of the most difficult complications of allo-HSCT [2]. In view of the major drawbacks of current treatments, a better therapy method is in urgent need for cGVHD. This study demonstrated therapeutic effects of oral administration of hydrogen-rich water on a scleroderma cGVHD mouse model. As to whether H_2_ has therapeutic effects on cGVHD through other mechanisms, further research is still needed.

## Data Availability

All data relevant to the study are included in the article.
